# Global Investigations of *Fusobacterium nucleatum* in Human Colorectal Cancer

**DOI:** 10.3389/fonc.2019.00566

**Published:** 2019-07-03

**Authors:** Seul A. Lee, Fang Liu, Stephen M. Riordan, Cheok S. Lee, Li Zhang

**Affiliations:** ^1^School of Biotechnology and Biomolecular Sciences, University of New South Wales, Sydney, NSW, Australia; ^2^Gastrointestinal and Liver Unit, Prince of Wales Hospital, University of New South Wales, Sydney, NSW, Australia; ^3^Discipline of Pathology, School of Medicine, Western Sydney University, Sydney, NSW, Australia; ^4^Faculty of Medicine and Health, Central Clinical School, University of Sydney, Sydney, NSW, Australia; ^5^Faculty of Medicine, South Western Sydney Clinical School, University of New South Wales, Sydney, NSW, Australia; ^6^Department of Anatomical Pathology, Liverpool Hospital, Sydney, NSW, Australia

**Keywords:** colorectal cancer, CRC, *Fusobacterium nucleatum*, *F. nucleatum*, tumorigenic mechanisms

## Abstract

Colorectal cancer (CRC) is the third most prevalent cancer and second in terms of mortality. Emerging evidence from recent studies suggests a potential role of *Fusobacterium nucleatum* in the development of CRC. In this article, we review studies from different geographical regions examining the association between *F. nucleatum* and CRC, the detection methods and the tumorigenic mechanisms. Furthermore, we discuss the potential clinical impact of *F. nucleatum* in CRC and suggest future study directions.

## Introduction

Colorectal cancer (CRC) is the third most commonly diagnosed cancer and second in terms of mortality (1). Over 95% of CRC are adenocarcinomas, and the majority develop via the adenoma-carcinoma sequence (2). Several risk factors are associated with CRC, such as genetic mutations on tumor suppressor genes and oncogenes, older age, diet, and chronic inflammation (3). In addition to these established risk factors, increasing evidence has linked CRC with some bacterial species in the gastrointestinal tract such as *Fusobacterium nucleatum*.

This article will review studies examining the association between *F. nucleatum* and CRC from different geographical regions. The potential clinical impact of *F. nucleatum* in CRC and the tumorigenic mechanisms of *F. nucleatum* in CRC will also be discussed.

## Fusobacterium Nucleatum

**Figure 1:**
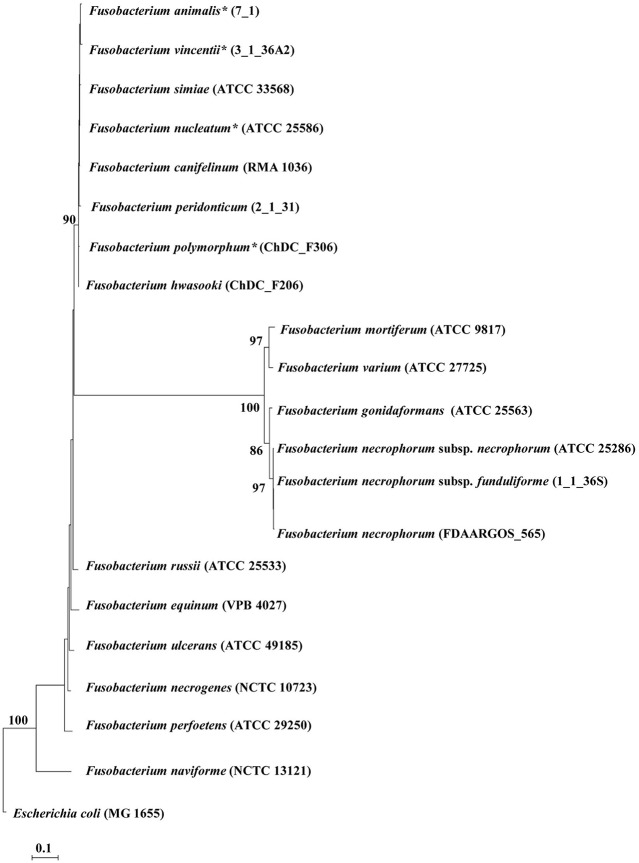
The phylogenetic tree generated based on 16S rRNA sequences of *Fusobacterium* species. The phylogenetic tree was generated using the maximum likelihood method. *Bootstrap* values were generated from 1,000 replicates and values of more than 70 were indicated. *Escherichia coli* MG1655 was included as an out group. Updated *Fusobacterium* species from previous subspecies are indicated by asterisks (*) (6). The 16S rRNA sequences of *Fusobacterium* species and *Escherichia coli* MG1655 were obtained from the National Center Biotechnology Information database.

*Fusobacterium* is a genus of the *Fusobacteriaceae* family, containing bacterial species isolated from both human and animal sources (4–6). *F. nucleatum*, a species of the *Fusobacterium* genus, previously contained four subspecies including *F. nucleatum* subsp. *nucleatum, F. nucleatum* subsp. *polymorphum, F. nucleatum* subsp. *vincentii*, and *F. nucleatum* subsp. *animalis* (7). A recent study by Kook et al. compared the genomes of the *F. nucleatum* subspecies with other *Fusobacterium* species and their study strongly suggested that these four subspecies should be reclassified as species *F. nucleatum, F. polymorphum, F. vincentii*, and *F. animalis*, respectively (6). The *Fusobacterium* genus currently contains 20 species and subspecies (Figure 1).

Within the *Fusobacterium* genus, most infections in humans are caused mainly by two species, *F. necrophorum* and *F. nucleatum*. *F. necrophorum* is a Gram-negative anaerobic bacterium with a rod shape and non-spore forming. *F. necrophorum* is found in the gastrointestinal tract of humans and animals and also in female urogenital tract and is the causative agent for Lemierre's syndrome (8–14). *F. nucleatum* is an oral Gram-negative anaerobe, with a spindle-rod shape, non-motile, and non-spore forming. *F. nucleatum* is associated with periodontitis (15–17). Furthermore, recent studies have linked *F. nucleatum* with human CRC.

## Studies Examining the Associations Between *F. nucleatum* and CRC and Pre-Cancerous Lesions From Different Geographical Regions

### Studies From Asia

Studies investigating *F. nucleatum* in CRC in Asia were reported from China and Japan. Using quantitative polymerase chain reaction (qPCR), Yan et al. examined the level of *F. nucleatum* relative to beta-actin in 280 stage III/IV Chinese CRC patients from Shanghai and found that the relative level of *F. nucleatum* in tumor tissues was significantly higher than the paired adjacent normal tissues (0.1092 ± 0.215 vs. 0.0245 ± 0.0553, *P* < 0.001; Table 1) (18). This study also found that a higher level of *F. nucleatum* was associated with tumor invasion, and patients with a low level of *F. nucleatum* had a significantly better cancer-specific survival and a disease-free survival (18).

**Table 1:** Studies examining the association between *Fusobacterium nucleatum* and CRC using colorectal tissues and fecal samples.

**References**	**Population**	**Samples (*N*)**	**Results**	**Detection methods**
Yan et al. (18)	China	CRC (280), adjacent normal tissues (280)	•*F. nucleatum* level was significantly higher in CRC tissues than in adjacent normal tissues (CRC vs. normal: 0.1092 ± 0.215 vs. 0.0245 ± 0.0553, *P* < 0.001).	Quantitative reverse transcription PCR using *F. nucleatum* specific primers
Li et al. (19)	China	CRC (101), adjacent normal tissues (101)	•The median abundance of *F. nucleatum* was significantly greater in the tumor samples [0.242 (0.178, 0.276)] than that in the matched normal controls [0.050 (0.023, 0.067)] (*P* < 0.001).	Fluorescent quantitative PCR using primer and probe sequences for *F. nucleatum* and fluorescence *in situ* hybridization (FISH) analysis
Wei et al. (20)	China	CRC (180), adjacent normal tissues (180)	•*F. nucleatum* between non-survival group and survival group (5.66 vs. 1.08%) and *F. nucleatum* (5.10 vs. 1.08%) exhibited a greater abundance in the recurrence group than in survival group, however, was not statistically significant. •High abundance of *F. nucleatum* was significantly correlated with the worse depth of invasion (*P* = 0.015).	Sequencing of V4 region of bacterial 16S rRNA gene
Yu et al. (21)	China	Cohort 1: CRC with recurrence (16), CRC without recurrence (15) Cohort 2: CRC with recurrence (48), CRC without recurrence (44) Cohort 3: CRC with recurrence (87), CRC without recurrence (86)	•In cohort 1, *Fusobacterium* was significantly enriched in recurrent tissues than non-recurrent tissues. Using qPCR, they also found that the relative abundance of *F. nucleatum* was significantly higher in recurrent CRC than non-recurrent tissues (*P* < 0.01). •In cohort 2, they also found that the relative abundance of *F. nucleatum* was significantly higher in tumor tissues, as compared to para-tumor tissues in both CRC patients with and without recurrence (*P* < 0.05). Furthermore, the *F. nucleatum* abundance in tumor tissues of CRC patients with recurrence was significantly higher than that in tumor tissues of CRC patients without recurrence (*P* < 0.01). •In cohort 3, *F. nucleatum* had a significantly higher abundance in recurrent CRC tissues than non-recurrent CRC tissues (*P* < 0.01).	Sequencing using 16S rRNA gene and quantitative real-time PCR using primers specific for *F. nucleatum*
Liang et al. (22)	China	Cohort 1: CRC (170), healthy controls (200)Cohort 2: CRC (33), healthy controls (36)	•The median abundance of *F. nucleatum* was significantly higher in CRC (0.0288 vs. 8.1E-6) than controls for Cohort 1 (*P* < 0.0001). For Cohort 2, abundance was also significantly higher in CRC than controls (*P* < 0.01). •The occurrence rate of *F. nucleatum* in CRC was 98.2% whereas in control 72% (*P* < 0.0001).	Duplex quantitative PCR targeting 16S rRNA gene (TaqMan method)
Wong et al. (23)	China	CRC (104), advanced adenoma (103) and healthy controls (102)	•In comparison to healthy controls, patients with CRC had a significantly higher abundance of *F. nucleatum* (132-fold, *P* < 0.001) •In comparison to healthy controls, patients with advanced adenoma had a significantly higher level of *F. nucleatum* (3.8-fold, *P* = 0.022).	Species specific quantitative real-time PCR (SYBR Green method)
Yamaoka et al. (24)	Japan	CRC (100), matched normal mucosa (72)	•The detection rates of *F. nucleatum* were 63.9% (46/72) in normal-appearing mucosal tissues and 75.0% (75/100) in CRC tissue samples and this was statistically insignificant. •The median copy number of *F. nucleatum* was 0.4 copies/ng DNA in the normal-appearing colorectal mucosa in patients with colorectal cancer and 1.9 copies/ng DNA in the colorectal cancer tissues (*P* = 0.0031).	Droplet digital PCR using primer targeting *F. nucleatum*
Ito et al. (25)	Japan	CRC (511), premalignant lesions: serrated lesions (343) and non-serrated adenomas (122)	•*F. nucleatum* positivity was significantly higher in CRCs (56%) than in premalignant lesions of any histological type (24% in hyperplastic polyps, 35% in sessile serrated adenomas, 30% in traditional serrated adenomas and 33% in non-serrated adenomas, *P* < 0.0001).	Quantitative PCR using primer specific for *F. nucleatum* (TaqMan method)
Suehiro et al. (26)	Japan	CRC stage I to IV (158), colorectal advanced adenoma/carcinoma *in situ* (19), colorectal non-advanced adenomas (11), healthy controls (60)	•The median copy numbers of *F. nucleatum* were 17.5 in the control group, 311 in the non-advanced adenoma group, 122 in the advanced adenoma/CIS group and 317 in the CRC group. •*F. nucleatum* level was significantly higher in the non-advanced adenoma group (*P* = 0.0147), the advanced adenoma/CIS group (*P* = 0.0060) and the CRC group (*P* < 0.0001) than the control group.	Droplet digital PCR using sequences of the *F. nucleatum* primer and probe set
Flanagan et al. (27)	Ireland, Czech Republic and Germany (European cohorts)	CRC (122), matched normal controls (122)	•In all three European cohorts, the abundance of *F. nucleatum* in tumor tissues were significantly higher as compared to the normal tissues (Czech Republic 266-fold *P* = 0.002, Germany 43-fold *P* = 0.0001 and Ireland 9-fold *P* = 0.006 respectively). •Significantly higher levels of *F. nucleatum* in tumor tissue (pooled cohorts RQ_Normal_ 2^−19^ vs. RQ_Tumour_ 2^−10^, *P* < 0.0001) were found. The average *F. nucleatum* levels were increased by over 45-fold in the pooled cohorts.	Quantitative real-time PCR using primers specific for *Fusobacterium* (SYBR Green method)
Bundgaard-Nielsen et al. (28)	Denmark	CRC (99), paired normal controls (99), CRA (96) and diverticular disease of the colon (104)	•*F. nucleatum* was detected higher in tumor tissues compared to adenoma (29.3 vs. 3.0%, *P* < 0.001). •Detection of *F. nucleatum* did not result in significant changes in survival or disease-free survival rates of patients within a 5-year period.	Quantitative real-time PCR using primers specific for *F. nucleatum* (SYBR Green method)
Eklöf et al. (29)	Sweden	CRC (39), dysplasia (134), controls (66)	•The abundance of *F. nucleatum* was significantly higher in fecal samples from patients with CRC than samples from dysplasia and controls (*P* < 0.001). •*F. nucleatum* was detected in 27 (69.2%) CRC, 27 (20.1%) dysplasia, and 15 (24.3%) controls (*P* < 0.001).	Quantitative real-time PCR using a FAM-labeled probe specific for *F. nucleatum* 16S rRNA gene
Russo et al. (30)	Italy	CRC (10), healthy controls (10)	•No significant difference between stool samples of healthy subjects of CRC patients was observed. However, it was found that *F. nucleatum* abundance was higher in saliva samples than stool samples in both healthy subjects (*P* < 0.002) and CRC patients (*P* < 0.01).	Quantitative real-time PCR using species-specific primers targeting 16S rRNA sequence
Castellarin et al. (31)	Canada	CRC (99), matched normal (99)	•RNA-Seq showed that *F. nucleatum* had the highest hits overall (21% of all alignments) and 9/11 subjects showed at least 2-fold higher read counts in tumor relative to corresponding control tissue. •qPCR showed that the mean overall abundance of *Fusobacterium* was found to be 415 times greater in the tumor samples than in the matched normal samples using qPCR (*P* < 2.52E-6).	RNA-Seq; quantitative PCR using primer/probe targeting *Fusobacterium* DNA (TaqMan method)
Mima et al. (32)	United States	CRC (598), adjacent non-tumor tissues (558)	•*F. nucleatum* was detected significantly higher in CRC tissues (13%, 76/598) than in non-tumor tissues (3.4%, 19/558), (*P* < 0.001). •In the 558 pairs of CRC and adjacent non-tumor tissues, the amount of *F. nucleatum* DNA in tumor tissues was significantly higher than in adjacent non-tumor tissues (*P* < 0.0001).	Quantitative PCR using primer targeting the *nus*G gene of *F. nucleatum* (TaqMan method)
Mima et al. (33)	United States	CRC (1102)	•*F. nucleatum* DNA was detected in 13% (138/1102) CRC tissues. The proportion of *F. nucleatum*-high colorectal cancers gradually increased from rectal cancers (2.5% 4/157) to cecal cancers (11% 19/178), with a statistically significant linear trend along with all subsites (*P* < 0.0001).	Quantitative PCR using primer targeting the *nus*G gene of *F. nucleatum* (TaqMan method)
Proença et al. (34)	Brazil	CRC (43), adjacent normal tissue (N-CRC 43), CRA (27), matched adjacent normal tissue (N-CRA 27)	•A significant increase in bacterial DNA was found for both CRA and CRC tissues compared to the respective normal adjacent tissues (*P* = 0.0002). The quantity of *F. nucleatum* was 24.84 times greater in CRC samples than in CRA samples (*P* < 0.0001).	Quantitative real-time PCR using *nus*G gene of *F. nucleatum* (TaqMan method)
Fukugaiti et al. (35)	Brazil	CRC (7), healthy controls (10)	•The level of *F. nucleatum* was significantly higher in fecal samples from patients with CRC than healthy patients (6.2 ±1.5 vs. 4.0 ± 1.5, *P* < 0.01).	Quantitative real-time PCR using species-specific primers (SYBR Green method)

*For studies from each country, studies using CRC tissues were listed first, followed by studies using fecal samples. CRC, colorectal cancer; CRA, colorectal adenoma; CIS, carcinoma in situ; RQ, relative quantification*.

A study by Li et al. using TaqMan probe-based qPCR examined *F. nucleatum* in cancer and adjacent normal tissues of 101 patients with CRC (19). They found that the median abundance of *F. nucleatum* in CRC tissues was 0.242 (0.178, 0.276), which was significantly higher than that in normal tissues, 0.05 (0.023, 0.067; *P* < 0.001) (19). In this study, Li et al. also found that the lymph node metastases was more frequently seen in patients with high abundance of *F. nucleatum* (19).

Wei et al. established libraries of 16S rRNA gene V4 region amplicon prepared from amplification of tumor samples from 180 Chinese patients with CRC stages I-IV from Qingdao (20). This study divided the patients into four groups including non-survival, recurrence, survival, and unknown. They found a greater abundance of *F. nucleatum* in the recurrence group than in the survival group and found that the high abundance of *F. nucleatum* significantly correlated with the worse depth of invasion (*P* = 0.015).

Yu et al. examined three cohorts of patients with CRC from a hospital in Shanghai, China (21). Fresh intestinal tissues were collected from patients of cohort 1 (31 patients) and subjected for microbiota analysis using 16S RNA sequencing, and it was found that *Fusobacterium* genus was enriched in recurrent CRC patients. Cohort 2 contained 44 recurrent CRC patients and 48 non-recurrent CRC patients, and formalin-fixed paraffin-embedded tissues were subjected for analysis using qPCR method. A significantly higher abundance of *F. nucleatum* in tumor tissues was observed, as compared to para-tumor tissues in both CRC patients with and without recurrence (*P* < 0.05). Furthermore, the abundance of *F. nucleatum* in tumor tissues of CRC patients with recurrence was significantly higher than that in tumor tissues of CRC patients without recurrence (*P* < 0.01). The higher level of *F. nucleatum* was strongly associated with shorter recurrence free survival. Cohort 3 contained 173 patients with CRC, and formalin-fixed paraffin-embedded tissues were used for qPCR analysis of *F. nucleatum*. Patients were divided into high and low *F. nucleatum* groups and an association between a higher level of *F. nucleatum* and shorter recurrence free survival was again found (21).

In addition to colonic tissue samples, fecal samples were also used to detect *F. nucleatum*. Liang et al. examined the abundance of five bacterial species in fecal samples of two independent Chinese cohorts using duplex qPCR targeting 16S rRNA gene (22). One cohort was from Hong Kong consisting of 170 patients with CRC and 200 healthy controls. The second cohort was from Shanghai consisting of 33 patients with CRC and 36 healthy controls. They found that the relative abundances of *F. nucleatum, Clostridium hathewayi*, and one unidentified species were higher in patients with CRC than in healthy controls for Hong Kong cohort (*P* < 0.0001) and the relative abundance of *F. nucleatum* was significantly higher in CRC patients than healthy controls for Shanghai cohort (*P* = 0.01) (22). Wong et al. examined the abundance of *F. nucleatum, Peptostreptococcus anaerobius* and *Parvimonas micra* relative to the total bacterial DNA in fecal samples collected from 104 patients with CRC, 103 patients with high grade adenoma and 102 controls in Hong Kong using qPCR SYBR Green method (23). This study found that the relative abundance of *F. nucleatum* in patients with CRC and advanced adenoma was 132-fold and 3.8-fold higher compared to controls (*P* < 0.001 and *P* = 0.022, respectively) (23).

The above studies examining the association between *F. nucleatum* and CRC in Chinese patients were based on *F. nucleatum* abundance. None of these studies reported the positive detection rate of *F. nucleatum* in the samples studied, it is therefore not clear how many patients with CRC were positive for *F. nucleatum* in the intestinal tissues or fecal samples.

A number of studies examining *F. nucleatum* in CRC were reported from Japan. Using digital PCR targeting *F. nucleatum*, Yamaoka at al. found that the median copy number of *F. nucleatum* in cancer mucosa of patients with CRC was significantly higher than that in normal-appearing mucosal tissues of these patients (1.9 copy number/ng vs. 0.4 copy number/ng DNA, *P* = 0.0031; Table 1) (24). After separating the CRC patients based on their disease stage, they found that the copy numbers of *F. nucleatum* in stage IV (not those in stages I to III) was significantly higher than that in the normal-appearing mucosa (*P* = 0.0016). This study also reported that *F. nucleatum* is commonly present in both mucosal tissues of CRC and normal-appearing tissues (75/100, 75% vs. 46/72, 63.95%) and the prevalence was not statistically different. Using *F. nucleatum* specific TaqMan qPCR method, Ito et al. examined tumor tissues from 511 Japanese CRC patients and 465 premalignant lesions including 343 serrated lesions and 122 non-serrated adenomas (25). They found that the *F. nucleatum* positivity was 56% in patients with CRC, which was significantly higher than in any of the premalignant lesions including 24% in hyperplastic polys, 35% in sessile serrated adenomas, 30% in traditional serrated adenomas (*P* < 0.0001). In contrast to other studies, the association between *F. nucleatum* and CRC reported by Ito et al. was based on prevalence rather than abundance. They also found that *F. nucleatum* was more frequently detected in premalignant lesions with high CpG Island methylator phenotype (CIMP) than in lesions with low or absent CIMP (46/108, 43 vs. 96/357, 27; *P* = 0.0023) (25).

Suehiro et al. also used digital PCR targeting *F. nucleatum* and found that in fecal samples, the median copy number of *F. nucleatum* was 317 in CRC group (158 patients), 122 in high grade adenoma/carcinoma *in situ* group (19 patients) and 311 in low grade adenoma group (11 patients). These were all significantly higher than the median copy number (17.5) in healthy controls (60 individuals) (26).

### Studies From Europe

A study from Flanagan et al. examined *F. nucleatum* in tumor and matched normal tissues from 122 patients with CRC from three European cohorts (Czech Republic, Germany, and Ireland; Table 1) (27). They found that in all three cohorts, the abundance of *F. nucleatum* in tumor tissues were significantly higher as compared to the normal tissues (*P* = 0.002, *P* = 0.0001 and *P* = 0.006 for Czech Republic, Germany, and Ireland, respectively) (27). They also examined *F. nucleatum* in 52 Irish patients with colorectal adenoma (CRA) and found a significantly higher level of *F. nucleatum* in adenoma tissues with high-grade dysplasia as compared to matched normal tissues (*P* = 0.015) (27).

A study from Denmark by Bundgaard-Nielsen et al. used tumor tissues, matched normal tissues, tissues from patients with adenoma and patients with diverticular disease to examine the association between *F. nucleatum* and CRC (Table 1) (28). By using qPCR, they have found that the abundance of *F. nucleatum* in tumor tissues were significantly higher as compared to adenoma tissues (29.3 vs. 3.0%, *P* < 0.001), however, when comparing to normal tissues, no significant difference was observed (28). This study also demonstrated that *F. nucleatum* did not affect the risk of death or the risk of developing new adenomas or CRC in patients with CRC, adenoma or diverticular disease (28).

Using fecal samples, Eklöf et al. from Sweden detected *F. nucleatum* in 69.2% of patients with CRC, 20.1% of patients with dysplasia and 24.3% of controls (*P* < 0.001; Table 1). They also found that *F*. *nucleatum* was significantly more abundant in fecal samples from patients with CRC than samples from dysplasia and controls (*P* < 0.001) (29). A study from Italy by Russo et al. did not find any significant difference between fecal samples from patients with CRC and healthy individuals (Table 1) (30). However, the sample size in that study was small (10 patients and 10 controls) (30).

### Studies From North America

Castellarin et al. compared the levels of *F. nucleatum* in tumor tissues and matched normal tissues from 99 Canadian patients and found a significantly higher level of *F. nucleatum* in tumor tissues as compared to normal tissues (*P* = 2.52E−6; Table 1) (31). The mean overall abundance of *F. nucleatum* was found to be 415 times greater in the tumor samples than in the matched normal tissues. They also found that patients with high-relative-abundance of *F. nucleatum* in tumor tissues relative to matched control tissues were significantly more likely to have regional lymph node metastases (*P* = 0.0035). Mima et al. from the United States examined *F. nucleatum* in tumor tissues of 598 patients with CRC and 558 adjacent non-tumor tissues; they found that both the positivity and the amount of *F. nucleatum* in tumor tissues were significantly higher than in non-tumor tissues (Table 1) (32). However, the detection rates of *F. nucleatum* in tumor and control tissues in the study from Mima et al. were 13 vs. 3.4%, which were dramatically lower than those reported from Asian countries. It is not clear whether this difference was due to different target genes used in the detection methods or ethnic background. In the following study, Mima et al. examined tumor tissues from 1,102 patients with CRC, found that the proportion of *F. nucleatum-*high cancers gradually increased from rectal cancers (2.5%; 4/157) to cecal cancers (11%; 19/178) (33). This is an interesting observation, given that *F. nucleatum* is an oral bacterium, it may have more opportunities to encounter with tumors at the cecum than those at the rectum.

### Studies From South America

A study from Brazil conducted by Proença et al. examined *F. nucleatum* in tumor tissues of 43 patients with CRC, 27 patients with CRA and matched adjacent normal tissues (Table 1) (34). A significant increase in *F. nucleatum* DNA was found in both CRA and CRC tissues compared to the respective normal adjacent tissues (*P* = 0.0002). Furthermore, the quantity of *F. nucleatum* was 24.84 times greater in CRC samples than in CRA samples (*P* < 0.0001). Fukugaiti et al. used fecal samples from seven patients with CRC and 10 healthy controls in Brazil to examine the level of *F. nucleatum* and found that *F. nucleatum* was detected at a significantly higher level in patients with CRC than healthy controls (6.2 ± 1.5 vs. 4.0 ± 1.5, *P* < 0.01) (35).

### Potential Clinical Implications of *F. nucleatum* in CRC

Two studies examined the use of *F. nucleatum* as a potential biomarker for detection of CRC: The significantly higher abundance of *F. nucleatum* in fecal samples of CRC patients when compared to healthy controls suggests that *F. nucleatum* is a potential biomarker for the early detection of CRC. In a study by Liang et al., DNA was first extracted from fecal samples collected from patients with CRC and healthy individuals, and duplex qPCR was performed using primers targeting *F. nucleatum* (22). Liang et al. reported a sensitivity of 77.7% and specificity of 79.5% of using *F. nucleatum* in fecal samples to discriminate CRC from controls (22). In the study from Wong et al., DNA was extracted from fecal samples of patients with CRC and high grade adenoma, and quantitative real-time PCR was performed using primers specific for *F. nucleatum* (23). Overall, the sensitivities of using *F. nucleatum* in fecal samples as a marker in detecting CRC and high grade adenoma were 73.1 and 15.5%, respectively, (23).

A number of studies also suggested that *F. nucleatum* is a potential therapeutic target for interfering with the progression of CRC: In the study from Yamaoka at al., the high abundance of *F. nucleatum* was found to be associated with stage IV of CRC, not the stages of I to III CRC (24). Yan et al. and Wei et al. found that *F. nucleatum* was adversely associated with CRC patient survival (18, 20). Castellarin et al. found that patients with high relative abundance of *F. nucleatum* in tumor tissues relative to matched control tissues were more likely to have regional lymph node metastases (31). These findings provide initial evidence that high levels of *F. nucleatum* may promote the progression of CRC. If these findings are confirmed, reducing *F. nucleatum* may be a potential strategy to alleviate the progression of CRC.

### Tumorigenic Mechanisms of *F. nucleatum* in CRC

**Figure 2:**
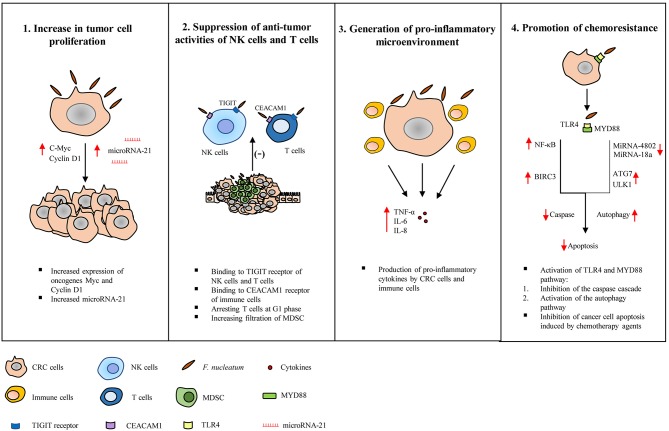
Tumorigenic mechanisms of *Fusobacterium nucleatum* in colorectal cancer. Studies have suggested the following tumorigenic mechanisms of *F. nucleatum* in colorectal cancer (CRC). (1) Increase in tumor cell proliferation: *F. nucleatum* increases the expression of oncogenes such as c-Myc and Cyclin D1 and microRNA-21, directly promoting tumor growth. (2) Suppression of the anti-tumor activity of natural killer (NK) and T cells: The activity of NK cells and T cells are suppressed by *F. nucleatum* through interaction with the T cell immunoglobulin and ITIM domain (TIGIT) receptor expressed on NK cells and T cells, binding and activating the carcinoembryonic antigen-related cell adhesion molecule (CEACAM) 1, arresting T cells at G1 phase and increasing the infiltration of myeloid-derived suppressor cells. (3) Generation of pro-inflammatory microenvironment: *F. nucleatum* induces pro-inflammatory cytokines production by cancer cells and immune cells. (4) Promotion of chemoresistance: *F. nucleatum* promotes chemoresistance to 5-fuorouracil (5-FU) by up-regulating baculoviral inhibitor of apoptosis protein repeat 3 (BIRC3) in CRC cells via Toll-like receptor 4 (TLR4)/Nuclear factor-kappa B (NF-κB) pathway, which results in inhibition of cancer cell apoptosis and reduced chemosensitivity to 5-FU. *F. nucleatum* also promotes chemoresistance by modulating autophagy. CRC cells infected with *F. nucleatum* activate the TLR4 and MYD88 innate immune signaling pathway, causing the loss of microRNAs miR-18a and miR-4802, and up-regulating autophagy elements, ULK1 and ATG7, which ultimately leads to inhibition of cancer cell apoptosis.

A number of studies have suggested the following mechanisms by which *F. nucleatum* may contribute to the development and progression of CRC, these mechanisms are summarized in Figure 2.

### Direct Promotion of the Growth of CRC Cells

Using CRC cell line models, several studies revealed that *F. nucleatum* was able to directly increase the growth of cancer cells. Rubinstein et al. showed that *F. nucleatum* stimulated the growth of several CRC cell lines such as HT-29 cells but did not stimulate the growth of the non-CRC cells such as HEK293 (36). By deletion of the *fadA* gene, this study demonstrated that FadA is a key virulence factor in promoting CRC cell growth by *F. nucleatum* (36). Rubinstein et al. also demonstrated that the cell-adhesion molecule E-cadherin acts as a receptor for FadA and binding of FadA to E-cadherin led to increased expression of transcription factor Nuclear factor-kappa B (NF-κB) and oncogenes such as c-Myc and Cyclin D1 (36). Another interesting finding from this study was that increased tumor growth and inflammatory responses caused by *F. nucleatum* were differentially regulated, further supporting a direct cancer promoting effect by *F. nucleatum* (36). A recent study conducted by the same group also reported that FadA can upregulate Wnt/β-catenin modulator Annexin A1 expression through E-cadherin (37).

Yang et al. demonstrated that *F. nucleatum* increases proliferation of CRC cell lines by up-regulating the expression of microRNA-21 (38). MicroRNA-21 functions as an oncogene, which suppresses the expression of tumor suppressor genes (38). This study further demonstrated that the increased expression of microRNA-21 by *F. nucleatum* was through binding of NF-κB to microRNA-21 promoter and the NF-κB pathway was activated through Toll-like receptor 4 (TLR4) (38).

### Suppression of Host Anti-tumor Immunity

Cytotoxic T cells and natural killer (NK) cells are the major effector cells to remove cancerous and pre-cancerous cells (39, 40). Shenker et al. demonstrated that *F. nucleatum* suppresses human T cell responses to mitogens and antigens by arresting cells in the G1 phase of the cell cycle (41, 42). Kaplan et al. showed that *F. nucleatum* outer membrane proteins Fap2 and RadD induce cell death in Jurkat cells (immortalized human lymphocytes) (43). Gur et al. further demonstrated that *F. nucleatum* prevents tumor cells from NK cell killing, via Fap2 protein binding to human inhibitory receptor T cell immunoglobulin and ITIM domain (TIGIT) on NK cells (44). They also showed that tumor-infiltrating lymphocytes expressed TIGIT and T cell activities were also inhibited by *F. nucleatum* via Fap2 (44). In addition, Fap2 also mediates *F. nucleatum* enrichment by interacting with Gal-GalNAc, a polysaccharide that is found to be overexpressed in CRC (45).

A recent study by Gur et al. demonstrated that an unidentified ligand of *F. nucleatum* inhibits NK cell and T cell function by binding to carcinoembryonic antigen-related cell adhesion molecule (CEACAM) 1 (46). CEACAM1 functions as an inhibitory receptor on various immune cell subsets (47). CEACAM1 is also expressed on the surface of tumors, and is considered to be a biomarker associated with tumor progression, metastasis and poor prognosis (48). It was previously shown that CEACAM1 expression is higher with more advanced stages of CRC, particularly in metastatic colon cancer, suggesting its role in CRC progression (49).

Mima et al. observed an inverse association between the amount of *F. nucleatum* and CD3^+^ T cell density in CRC tissues (32). Kostic et al. showed that *F. nucleatum* fed APC^Min/+^ mice had a significantly higher number of colonic tumors as compared to control groups without causing enteritis (50). Furthermore, this study found increased filtration of CD11b^+^ myeloid-derived suppressor cells (MDSC) in the tumors of APC^Min/+^ mice fed with *F. nucleatum* as compared to control APC^Min/+^ mice, while the numbers of CD3^+^/CD4^+^ and CD3^+^/CD8^+^ T lymphocytes were not affected. MDSCs are known to suppress T cells and play an important role in promoting tumor progression (51).

Microsatellite instability (MSI) is a form of genetic instability caused by alterations in the DNA mismatch repair system and ~15% of colorectal cancers display MSI (52). A number of studies have shown the association between high MSI and a great amount of *F. nucleatum* in CRC tissues (32, 50, 53, 54). A recent study by Hamada et al. has shown an association between *F. nucleatum* with the immune response to CRC and tumor MSI status (53). The study found that the presence of *F. nucleatum* was negatively associated with TIL in MSI-high tumors whereas, positively associated with TIL in non–MSI-high tumors (53). Hence, *F. nucleatum* may promote immune evasion in MSI-high colorectal carcinomas by inducing suppressive effects on adaptive anti-tumor immune responses in MSI-high CRC. However, further studies are required to discover the exact mechanism of how *F. nucleatum* affects the epigenetic changes in CRC. Collectively, these studies have shown that colonization of *F. nucleatum* in CRC and pre-cancerous tissues may reduce host ability in eliminating cancer and precancerous cells by direct inhibition of immune effector cells or modulating tumor-immune microenvironment.

### Inducing Inflammation

It is well-known that chronic inflammation induced by persistent microbes, such as *Helicobacter pylori* and hepatitis B and C, promotes cancer development and progression (55, 56). Chronic inflammation contributes to tumorigenesis through multiple mechanisms such as causing DNA damage, increasing mutation rates and damaging repair enzymes (57). Kostic et al. found that tumor tissues in *F. nucleatum* fed APC^Min/+^ mice had increased expression of cytokines such as tumor necrosis factor (TNF)-α, interleukin (IL)-6 and IL-8, which were similar to what has been observed in human CRC (50). Several other studies also showed that *F. nucleatum* induced production of pro-inflammatory cytokines and activation of NF-κB pathway (58, 59). NF-κB activates genes that control cell survival, proliferation and angiogenesis (60). Collectively, these data suggest that *F. nucleatum* may contribute to CRC development and progression via generating a pro-inflammatory microenvironment.

### Promoting Chemoresistance

Studies have shown that gut microbiota can influence the efficacy of anti-tumor immunotherapy drugs and have an impact on the treatment of CRC (61–66). Using colon cancer cell lines, Zhang et al. demonstrated that *F. nucleatum* infection reduced chemosensitivity of CRC cells to 5-fluorouracil (5-FU), a chemotherapeutic drug commonly used in treating patients with advanced CRC (67). This study showed that *F. nucleatum* infection up-regulated baculoviral inhibitor of apoptosis protein repeat 3 (BIRC3), a member of the inhibitor of apoptosis proteins (IAPs), which inhibits apoptosis by directly inhibiting the caspase cascade (67–69). IAPs are also known to promote the survival of tumor cells and induce chemoresistance (70, 71). Studies have reported that overexpression of BIRC3 is associated with chemoresistance in the treatment of CRC (72, 73). Using the same cell line models, Zhang et al. further demonstrated that BIRC3 expression in CRC cells infected with *F*. *nucleatum* is regulated via TLR4/NF-κB pathway and reduced the chemosensitivity of CRC cells to 5-FU. Consistent with studies using the cell line models, *F. nucleatum* also induced chemoresistance of CRC cells in response to 5-FU in a mouse model. It was found that a high abundance of *F. nucleatum* correlated with chemoresistance in advanced CRC patients receiving 5-FU-based chemotherapy, suggesting that *F. nucleatum* may be used as a target to improve the therapeutic response of advanced CRC patients receiving 5-FU-based chemotherapy (67).

Yu et al. has found that *F. nucleatum* promoted chemoresistance to CRC cells by modulating autophagy (21). By using colon cancer cell lines, they showed that *F. nucleatum* induced CRC resistance to chemotherapeutic drugs, Oxaliplatin and 5-FU, via TLR4 and MYD88 innate immune signaling pathway (21). By acting on this pathway, *F. nucleatum* downregulated microRNAs miR-18a and miR-4802, and upregulated autophagy elements such as ULK1 and ATG7, leading to inhibition of cancer cell apoptosis and enhanced chemoresistance (21). Tissues from patients with CRC and normal subjects were also examined, which showed that a higher *F. nucleatum* abundance, upregulated expression of ULK1, and ATG7 and loss of miR18a and miRA-4802 were associated with disease recurrence (21). Collectively, the study suggests that *F. nucleatum* plays a role in chemoresistance.

## Discussion and Future Work

Studies from different geographical regions reported the association between *F. nucleatum* and CRC using molecular detection methods. In most of these studies, the association was based on comparison of *F. nucleatum* abundance in CRC tissue sample with matched normal tissues. A few studies also reported the association based on the prevalence of *F. nucleatum* in CRC tissues or fecal samples as compared to controls. The prevalence of *F. nucleatum* in CRC tissues reported varied greatly, while the study by Mima et al. from the United States reported the detection rate of 13%, a study by Yamaoka et al from Japan detected *F. nucleatum* in 75% CRC tissues (24, 32, 33). It is not clear whether such a great difference was due to different detection methods used. Furthermore, a study from Denmark did not find an association between *F. nucleatum* and CRC and the CRC progression (28). A standardized method is required to generate reproducible and consistent data as well as to clarify whether the differences between the detection rate of *F. nucleatum* is due to ethnicity.

A higher level of *F. nucleatum* in fecal samples of patients with CRC and adenoma was detected as compared to healthy controls (23, 26). This may be due to increased shedding of *F. nucleatum* from the neoplastic tissues. Another possibility is that more *F. nucleatum* has been transported from the oral cavity to the intestinal tract in patients with CRC than healthy controls. This view is supported by a recent study by Komiya et al. showing that patients with CRC had identical strains in their colorectal cancer and oral cavity (74). However, examinations of the abundance of *F. nucleatum* in saliva samples of patients with CRC and healthy controls reported inconsistent results. While Guven et al. reported a significantly higher amount of *F. nucleatum* in saliva samples of patients with CRC than in controls, Russo et al. did not observe such a difference (30, 75). In contrast, Russo et al. detected a high prevalence of *F. nucleatum* in saliva samples of both patients with CRC (*P* < 0.01) and healthy controls (*P* < 0.002) than in stool samples showing that *F. nucleatum* is commonly present in the human oral cavity (30). Thus, whether oral *F. nucleatum* directly contributes to the increased *F. nucleatum* in the fecal samples of CRC remains to be investigated.

Several studies also suggest that high levels of *F. nucleatum* may promote the progression of CRC (27, 36, 37). If these mechanisms are confirmed, reducing or eliminating *F. nucleatum* may be a potential strategy to alleviate the progression of CRC, and the use of antibiotics might be one of the therapeutic interventions. However, a study from Cao et al. showed that the long-term use of antibiotics is associated with an increased risk of developing colorectal adenoma, although this study did not specify the antibiotics that were being used (76). Furthermore, whether these antibiotics could change the composition of microbiota in the gastrointestinal tract, and how this may affect the progression of CRC is unclear. Therefore, further studies are required to evaluate the efficacy of different antibiotics in reducing or eliminating *F. nucleatum* from the gastrointestinal tract and their relationship with CRC development and progression. Another possible therapeutic strategy is through microbiota manipulation. Bacterial species that are positively associated with a better prognosis of CRC in patients with a low level of *F. nucleatum* could be considered as potential candidates, which may be used as probiotic bacterial species to reduce the level of *F. nucleatum*. The use of fecal *F. nucleatum* as a biomarker for detection of CRC was suggested by two studies, which remains to be verified by additional studies (22, 23).

In conclusion, despite recent interesting findings in the field of *F. nucleatum* and CRC, whether this bacterium can be used as a CRC detection marker or a therapeutic target for intervention in tumor progression still need further investigation. Furthermore, *F. nucleatum* strains should be isolated from patients with CRC and healthy controls to investigate whether there are tumor-associated virulence factors.

## Author Contributions

SL played a major role in writing the manuscript. LZ, FL, SR, and CL provided important feedback and helped in editing the manuscript. All authors have approved the final version of the manuscript.

### Conflict of Interest Statement

The authors declare that the research was conducted in the absence of any commercial or financial relationships that could be construed as a potential conflict of interest.
